# Overexpression of Sly-miR398b Compromises Disease Resistance against *Botrytis cinerea* through Regulating ROS Homeostasis and JA-Related Defense Genes in Tomato

**DOI:** 10.3390/plants12132572

**Published:** 2023-07-07

**Authors:** Yuanyuan Liu, Yiren Yu, Shihong Fei, Yuxin Chen, Yunmin Xu, Zhujun Zhu, Yong He

**Affiliations:** Collaborative Innovation Center for Efficient and Green Production of Agriculture in Mountainous Areas of Zhejiang Province, Key Laboratory of Quality and Safety Control for Subtropical Fruit and Vegetable, Ministry of Agriculture and Rural Affairs, College of Horticulture Science, Zhejiang A&F University, Hangzhou 311300, China; yyliu@zafu.edu.cn (Y.L.); 2019101032007@stu.zafu.edu.cn (Y.Y.); 2020101031003@stu.zafu.edu.cn (S.F.); 202001070211@stu.zafu.edu.cn (Y.C.); xuyunmin@zafu.edu.cn (Y.X.)

**Keywords:** tomato, miR398, reactive oxygen species, MeJA, *Botrytis cinerea*

## Abstract

MicroRNAs (miRNAs) have been shown to be critical components in plant immunity. MicroRNA398 (miR398) is a highly conserved miRNA in all land plants and plays crucial roles in diverse biotic stress responses. However, the role of miR398 has not yet been characterized in tomato resistance against *Botrytis cinerea*. In this report, the transcript levels of sly-miR398b were strongly decreased in *B. cinerea*-infected leaves and the overexpression of sly-miR398b resulted in enhanced susceptibility. The attenuated expression of *cytosol Cu/Zn-SOD* (*CSD1*), *chloroplast Cu/Zn-SOD* (*CSD2*), and *guaiacol peroxidase* (*GPOD*), as well as the decreased activities of superoxide dismutase (SOD) and GPOD, collectively led to increased hydrogen peroxide (H_2_O_2_) accumulation in sly-miR398b overexpressing plants. Furthermore, sly-miR398b was induced by methyl jasmonate (MeJA) treatment. The overexpression of sly-miR398b suppressed the expression of *TomLoxD*, *LapA*, and *PR-STH2* in response to *B. cinerea* and MeJA treatment. Our data demonstrate that sly-miR398b overexpression negatively regulates the resistance to *B. cinerea* in tomato by inducing the accumulation of reactive oxygen species (ROS) and downregulating the expression of MeJA-responsive defense genes.

## 1. Introduction

Tomato (*Solanum lycopersicum*) is the most important vegetable crop worldwide (Food and Agriculture Organization of the United Nations; http://faostat.fao.org/site/339/default.aspx (accessed on 20 January 2022)). With the improvement in people’s living standards and the increasing demand for fruits and vegetables, the annual supply of fruits and vegetables is becoming more and more important. Due to low temperatures, high humidity, low light, and other reasons, overwinter-grown tomatoes and greenhouse-grown tomatoes are prone to *Botrytis cinerea* infection. *Botrytis* species are responsible for gray mold rot and cause devastating diseases and significant crop losses in over 1000 plant species, including tomato, grape, and strawberry [1]. A large number of studies have shown that most cultivated tomato varieties are susceptible to *B. cinerea* [2], causing about 20–40% losses in tomato crops, which could rise to 50–70% during the disease epidemic periods [3].

Plant pathogens could be classified into necrotrophs, hemi-biotrophs, and biotrophs based on their lifestyles. In response to different invasion modes of pathogens, plants have evolved complex and fine defense response pathways controlled by plant hormones such as jasmonic acid (JA) and salicylic acid (SA). It is generally believed that plants use the SA-mediated signaling pathway to defend against biotrophic and hemibiotrophic pathogens, and use the JA-mediated signaling pathway to defend against necrotrophic pathogens such as *B. cinerea*. The defense responses mediated by SA and JA are generally antagonistic [4]. A large amount of evidence supports the JA signaling pathway playing an important role in regulating plant defense responses to *B. cinerea* [4]. In Arabidopsis and tomato, related mutants, which are defective in JA biosynthesis and signaling, are more sensitive to *B. cinerea* infection. The *coronatine insensitive 1* (*coi1-1*) and *jasmonic acid-insensitive1* (*jai1*) mutants, which harbor a mutation in the JA receptor-encoding gene *COI1* in Arabidopsis and tomato, respectively, show larger lesion areas than wild-type (WT) after inoculation with *B. cinerea* [5,6]. The tomato mutant *spr8*, which harbors a dominant negative mutation in *Tomato lipoxygenase D* (*TomLoxD*) and is defective in JA biosynthesis, is more susceptible than WT plants to *B. cinerea* infection [7]. Methyl jasmonate (MeJA), a volatile methyl ester of JA, is an important active form of JA that confers protection against fungal pathogens [8]. Exogenous MeJA application can effectively suppress gray mold disease in tomato by promoting the activities of antioxidant enzymes and upregulating the expression of a series of defense-related genes [9]. A whole-genome transcriptome analysis revealed that JA could not only induce the expression of the JA biosynthetic genes, such as *TomLoxD*, but also upregulate several defense genes, including *Leucine aminopeptidase A* (*LapA*) and *Pathogenesis-related salt tolerance homolog 2* (*PR-STH2*) [5,7,10].

In addition to plant hormones, reactive oxygen species (ROS) have been demonstrated to play an important role in plant–pathogen interactions [11]. ROS in plants usually include singlet oxygen (^1^O_2_), superoxide anion (O_2_^•−^), hydroxyl radical (OH^•^), and H_2_O_2_. The infection of plants by *B. cinerea* is characterized by the production of phytotoxins and cell wall degrading enzymes and an oxidative burst that finally leads to plant cell death [12]. Upon inoculation with *B. cinerea*, ROS, especially hydrogen peroxide (H_2_O_2_), is rapidly generated and accumulated within 12 h at the inoculation site [12]. As *B. cinerea* is a necrotrophic fungus, these ROS could facilitate the invasion and spread of the pathogen in the dead host tissue [13,14,15,16]. Plasma membrane-localized NADPH oxidases (respiratory burst oxidase homologs, Rbohs) transport electrons through membranes, reducing oxygen to O_2_^•−^ using NADPH as an electron donor [17]. Then, SOD catalyzes the conversion of O_2_^•−^ into H_2_O_2_. Subsequently, H_2_O_2_ is detoxified into H_2_O and O_2_ by catalase (CAT), GPOD, and ascorbate peroxidase (APX) [18].

MicroRNAs (miRNAs), a group of 20–24-nucleotide-long, non-coding RNAs, play regulatory roles at the transcriptional levels by guiding target mRNA for degradation or post-transcriptionally via translational inhibition by base pairing [19,20,21,22,23]. Studies have shown that miRNAs are involved in coordinating plant–pathogen interactions [24,25,26,27]. For example, miR398 in *A. thaliana* is downregulated by *Pseudomonas syringae* pv. tomato [28] and *A. thaliana* miR398a-5p is upregulated upon *P. capsici* infection [29]. miR398 is a conserved miRNA first identified in Arabidopsis [30,31]. Studies have revealed that miR398 targets *cytosol Cu/Zn-SOD* (*CSD1*), *chloroplast Cu/Zn-SOD* (*CSD2*), and *Copper chaperone for SOD* (*CCS1*) to regulate ROS concentration and plant disease resistance against multiple pathogens, including bacteria, fungi, and viruses [31,32,33,34,35]. In Arabidopsis, the overexpression of miR398 enhances plant susceptibility to *P. syringae* pv. tomato DC3000 by suppressing *CSD1* and *CSD2* [28,32,36]. In barley, a reduced miR398 amount and increased SOD1 accumulation are associated with enhanced resistance against powdery mildew [35]. In rice, however, Osa-miR398b overexpression enhances resistance against *Magnaporthe oryzae* by reducing the abundance of CSD1, CSD2, Superoxide DismutaseX (SODX), and Copper Chaperone for Superoxide Dismutase (CCSD) [37]. In *Nicotiana benthamiana*, Nb-miR398 negatively regulates plant immunity to the *Bamboo mosaic virus* (BaMV) and downregulates the *NbCSD1* and *NbCSD2* genes [33]. These findings indicate that the miR398-SOD module plays important roles in regulating plant resistance against pathogens; however, the fact that higher ROS (presumably, given the lower enzymatic antioxidants) might lead to higher resistance in one pathosystem but not in the other can possibly be due to connections with different pathogen lifestyles. Whether miR398 participates in regulating tomato defense against *B. cinerea* and its role in tomato–*B. cinerea* interaction remains unknown, and needs to be verified experimentally without simply relying on results obtained in different pathosystems. In addition, given the role of the JA pathway in this interaction, a possible molecular connection between miR398 and JA signaling also seems worthy of investigation.

The aim of this work was to characterize the function of sly-miR398b in tomato defense against *B. cinerea*. We first analyzed the expression in response to infection and then investigated the effects of sly-miR398b overexpression on resistance. In addition, the activities and gene expression of defense enzymes, as well as the transcripts of JA-responsive genes, were also measured. Our results in this report extend the knowledge of the role of miR398 in tomato–*B. cinerea* interactions.

## 2. Results

### 2.1. Effects of B. cinerea Infection on the Expression of Pri-miR398b and Sly-miR398b

**Figure 1:**
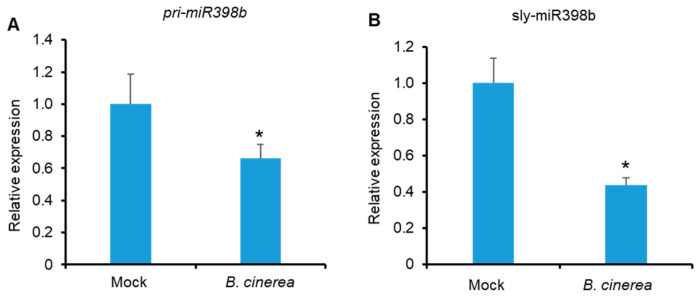
Effects of *B. cinerea* infection on *pri-miR398b* (**A**) and sly-miR398b levels (**B**). Four-week-old tomato plants were spotted with a 5-µL spore suspension (10^6^ spores/mL) of *B. cinerea* or with 1% Sabouraud Maltose Broth (mock). Leaf samples were collected 48 h after inoculation. For (**A**,**B**), relative levels were calculated by comparing with the corresponding values of uninfected leaves (mock). *SlACTIN* was used as a reference gene for *pri-miR398b* in qRT-PCR. Tomato U6 was used as a reference gene for sly-miR398b in a stem-loop qRT-PCR assay. Values presented are the means ± SD from three biological replicates. Asterisks indicate significant differences detected using Student’s *t* test (*, *p* < 0.05) when compared with uninfected leaves (mock).

To elucidate the role of sly-miR398b in plant resistance, we first assessed *MIR398b* expression profiles by quantifying *pri-miR398b* in tomato leaves upon *B. cinerea* infection via a quantitative reverse transcription PCR (qRT-PCR) assay. We also quantified the effects of mature sly-miR398b in tomato leaves upon *B. cinerea* infection via a stem-loop qRT-PCR assay. As the results showed that the amounts of *pri-miR398b* and sly-miR398b in tomato leaves decreased during infection (Figure 1A,B), we thus postulated that sly-miR398b might participate in regulating tomato immunity against *B. cinerea*.

### 2.2. Overexpression of Sly-miR398b Enhances Tomato Susceptibility to B. cinerea

**Figure 2:**
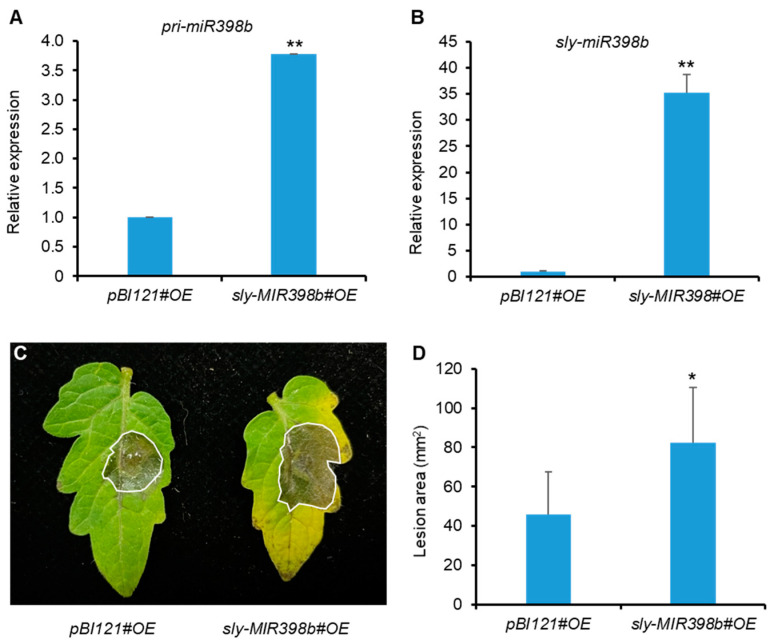
Overexpression of sly-miR398b enhances susceptibility to *B. cinerea* in tomato plants. (**A**) RT-qPCR assays show the amounts of *pri-miR398b* in *PBI121#OE* and *sly-MIR398b#OE* plants. (**B**) Stem-loop RT-qPCR assays show the expression of sly-miR398b in *PBI121#OE* and *sly-MIR398b#OE* plants. Total RNAs were extracted from 18-day-old seedlings. *SlACTIN* was used as reference gene for *pri-miR398b*. Tomato *U6* was used as reference gene for sly-miR398b. Values presented are the means ± SD from three biological replicates. ** above the columns indicate significant differences at *p* < 0.01 level. (**C**,**D**) Response of *PBI121#OE* and *sly-MIR398b#OE* plants to *B. cinerea* infection. Inoculation was carried out by dropping spore suspensions (10^6^ spores/mL) on detached leaves of four-week-old plants. Photographs (**C**) were taken and the lesion areas (**D**) were analyzed 48 h after inoculation. Error bars represent the SE from six replicates (n = 6). Asterisks indicate a significant difference from the wild type according to Student’s *t* test at * *p* < 0.05.

To investigate whether sly-miR398b would affect the immune function of tomato regarding *B. cinerea*, we obtained sly-miR398b-overexpression (*sly-MIR398b#OE*) transgenic plants and *pBI121#OE* control plants in the cultivar Micro-Tom [38]. The transcript level of *pri-miR398b* and sly-miR398b in *sly-MIR398b#OE* was noticeably higher than that in the *pBI121#OE* control plants (Figure 2A,B). The detached leaves from four-week-old tomato plants were incubated with *B. cinerea* spore suspensions for 48 h. The results indicated that *B. cinerea* infection leads to significantly larger necrotic lesions in *sly-MIR398b#OE* plants than in the *pBI121#OE* control plants (Figure 2C,D). Together, these results substantiate sly-miR398b overexpression negatively regulating tomato resistance against *B. cinerea* infection.

### 2.3. Overexpression of Sly-miR398b Results in the Accumulation of H_2_O_2_

**Figure 3:**
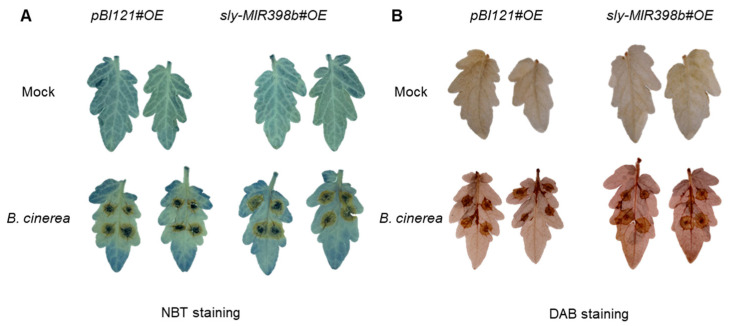
Overexpression of sly-miR398b increases *B. cinerea*-induced ROS accumulation. (**A**) O_2_^−^ accumulation. (**B**) H_2_O_2_ accumulation. For the detection of the accumulation of O_2_^−^ and H_2_O_2_, detached leaves were stained with nitroblue tetrazolium (NBT) and 3,3-diaminobenzidine (DAB), respectively. Leaves from *PBI121#OE* and *sly-MIR398b#OE* plants were harvested 48 h after inoculation with a 5-µL spore suspension (10^6^ spores/mL) of *B. cinerea* or with 1% Sabouraud Maltose Broth (mock).

Previous studies have demonstrated that miR398b mediates plant immunity by regulating ROS homeostasis [39,40]. To analyze the effects of sly-miR398b overexpression on ROS homeostasis, the concentration of O_2_^•−^ and H_2_O_2_ in *B. cinerea* -inoculated tomato leaves were compared between *pBI121#OE* control plants and *sly-MIR398b#OE* plants. As the results show in Figure 3A, no difference was observed in both the untreated and treated leaves of *pBI121#OE* control plants and *sly-MIR398b#OE* plants in terms of O_2_^•−^. However, incubation with *B. cinerea* upregulated the level of H_2_O_2_ (Figure 3B). The H_2_O_2_ content in the *sly-MIR398b#OE* plants was significantly higher than that in the *pBI121#OE* control plants at 48 h after *B. cinerea* infection, while there was no difference detected between the uninfected leaves of either genotypes (Figure 3B).

### 2.4. Overexpression of Sly-miR398b Decreases the Activities of Antioxidant Enzymes and the Relative Expression of Antioxidant Genes

**Figure 4:**
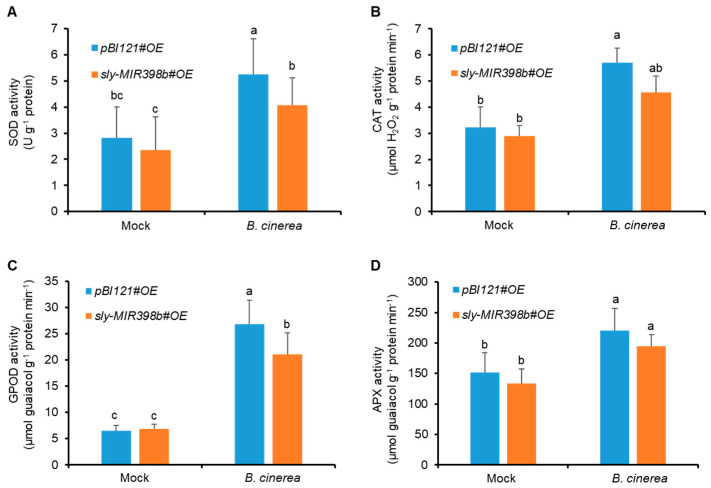
Effects of sly-miR398b overexpression on *B. cinerea*-induced activities of antioxidant enzymes 48 hpi. (**A**), SOD. (**B**), CAT. (**C**), GPOD. (**D**), APX. Each value represents a mean ± SE of seven biological replicates. Different letters above the bars indicate significant differences (*p* < 0.05, one-way ANOVA) according to the least significant difference (LSD) test.

To further investigate the effects of sly-miR398b overexpression on ROS homeostasis in tomato plants, activities of SOD, CAT, GPOD, and APX in *pBI121#OE* control plants and *sly-MIR398b#OE* plants were examined. The activities of SOD, CAT, GPOD, and APX in *sly-MIR398b#OE* plants were decreased compared to *pBI121#OE* control plants, with values 28.21%, 24.66%, 26.88%, and 13.61% lower than those in *pBI121#OE* control plants 48 hpi (hours post inoculation), respectively (Figure 4). However, SOD and GPOD activities in *sly-MIR398b#OE* plants were significantly lower than those in *pBI121#OE* control plants at 48 hpi (Figure 4C).

**Figure 5:**
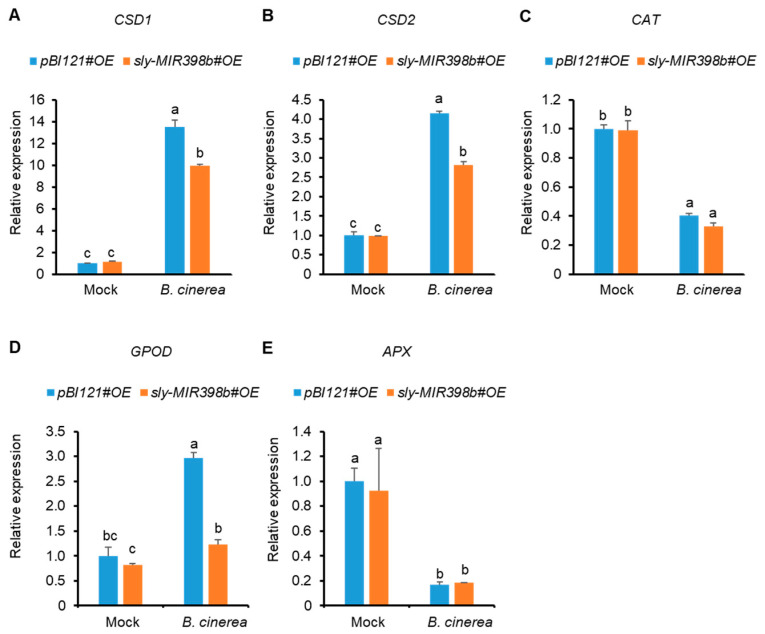
Effects of sly-miR398b overexpression on *B. cinerea*-induced relative expressions of antioxidant genes 48 hpi. (**A**), *CSD1*. (**B**), *CSD2*. (**C**), *CAT*. (**D**), *GPOD*. (**E**), *APX*. *SlACTIN*, *SlEF1α*, and *SlSAND* were used as reference genes. Each value represents a mean ± SD of three biological replicates. Different letters above the bars indicate significant differences (*p* < 0.05, one-way ANOVA) according to the least significant difference (LSD) test.

In uninfected tomato leaves, the relative transcript levels of antioxidant genes including *CSD1*, *CSD2*, *CAT*, *GPOD*, and *APX* were not affected in *sly-MIR398b#OE* plants (Figure 5). Moreover, compared to *pBI121#OE* control plants, the transcript levels of *CAT* and *APX* in *sly-MIR398b#OE* plants showed no difference 48 hpi, but both CAT and APX transcripts were decreased significantly by infection (Figure 5C,E). However, the transcript levels of *CSD1*, *CSD2*, and *GPOD* were increased significantly by infection, and they were significantly lower in *sly-MIR398b#OE* plants than those in *pBI121#OE* control plants 48 h after *B. cinerea* infection (Figure 5A,B,D).

### 2.5. Effects of Sly-MIR398b Overexpression on JA-Related Defense Genes

**Figure 6:**
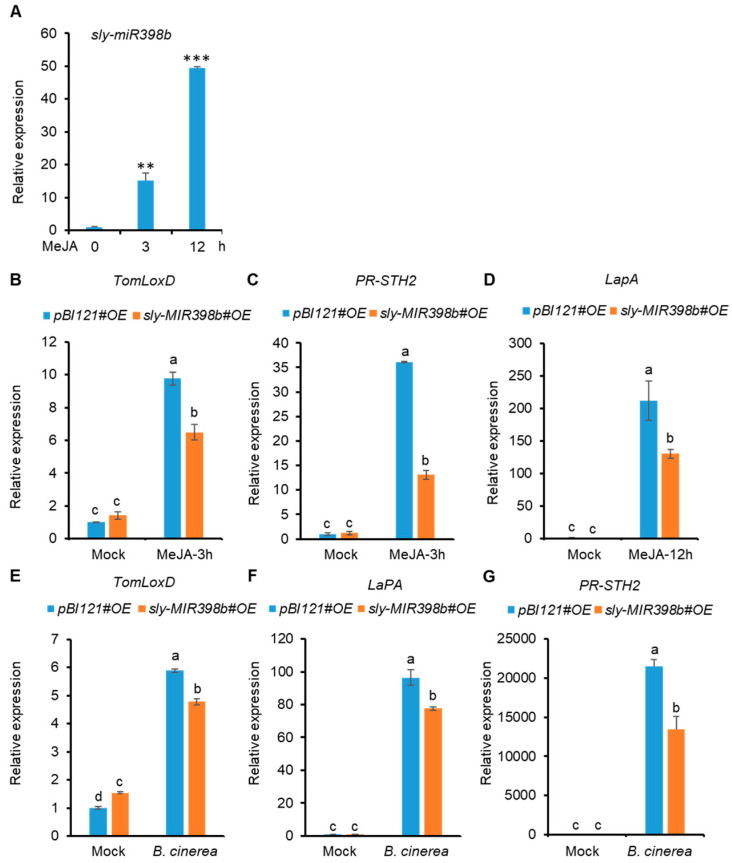
sly-miR398b negatively regulates JA-responsive defense genes. (**A**) Accumulation patterns of mature sly-miR398b in response to MeJA. The *U6* was used as reference gene for sly-miR398b. (**B**–**D**) RT-qPCR assays of *TomLoxD* (**B**), *LaPA* (**C**), and *PR-STH2* (**D**) transcripts in *PBI121#OE* and *sly-MIR398b#OE* plants in response to MeJA treatment. For MeJA treatment, 18-day-old seedlings of the indicated genotypes with two fully expanded leaves were exposed to MeJA vapor for the indicated times (hours) before extracting total RNAs for RT-qPCR assays. (**E**–**G**) RT-qPCR assays of *B. cinerea*-induced relative transcript amounts of *TomLoxD* (**E**), *LaPA* (**F**), and *PR-STH2* (**G**) in *PBI121#OE* and *sly-MIR398b#OE* plants. Leaves from *PBI121#OE* and *sly-MIR398b#OE* plants were harvested 48 h after inoculation with a 5-µL spore suspension (10^6^ spores/mL) of *B. cinerea* or with 1% Sabouraud Maltose Broth (mock). For (**B**–**G**), *SlACTIN* was used as a reference gene. Each value represents a mean ± SD of three biological replicates. For (**A**), asterisks indicate significant differences from *PBI121#OE* plants according to Student’s *t* test at ** *p* < 0.01 and *** *p* < 0.001. For (**B**–**G**), different letters above the bars indicate significant differences (*p* < 0.05, one-way ANOVA) according to the least significant difference (LSD) test.

Jasmonic acid (JA) signaling is believed to have a pivotal role in plant defense against necrotrophic pathogens [4,6]. Thus, we explored whether sly-miR398b affected tomato resistance to *B. cinerea* by influencing JA signaling. First, the accumulation pattern of sly-miR398b in response to MeJA was assessed. Stem loop RT-qPCR assays showed that the abundance of sly-miR398b was upregulated 3 and 12 h after treatment with MeJA (Figure 6A). Second, MeJA-induced transcript levels of *TomLoxD*, *LapA*, and *PR-STH2* were decreased in *sly-MIR398b#OE* plants compared with *pBI121#OE* control plants (Figure 6B–D).

To further investigate the regulatory mechanism of sly-miR398b-mediated susceptibility to *B. cinerea*, the transcripts of JA-inducible defense genes were quantified 48 h after *B. cinerea* infection. Results showed that, compared to *pBI121#OE* control plants, the abundance of *TomLoxD*, *LapA*, and *PR-STH2* transcripts in *sly-MIR398b#OE* plants were significantly decreased 48 hpi (Figure 6E–G). These results indicated that the overexpression of *sly-MIR398b* hampered the expression of JA-responsive defense genes in response to *B. cinerea* infection.

## 3. Discussion

miRNAs have been found to play important roles in response to various biotic and abiotic stresses in plants, including biotic stresses (bacteria, fungi, viruses, insects, nematodes, etc.) and abiotic stresses (salinization, drought, low/high temperature, nutrient deficiencies, etc.) [41]. Uncovering the mechanisms mediated by miRNAs in these stress responses will help us to utilize miRNA-mediated defense mechanisms and promote resistance breeding in tomato.

miR398 is a highly conserved miRNA and has been demonstrated to play important roles in both plant development and stress responses [39]. As computationally predicted by the base-pairing principle in Arabidopsis and rice, firstly, the target genes of miR398 were *CSD1* and *CSD2*, which are responsible for scavenging ROS [31,32,33,42]. Interestingly, miR398 was further shown to regulate CSD2 at the protein level through translational inhibition [22,23]. A growing body of research suggests that the miR398-CSD regulation module is implicated in biotic stress responses [39]. Here, sly-miR398b overexpression was used to investigate how sly-miR398b influences tomato resistance against *B. cinerea*. Results showed that overexpression significantly enhances susceptibility. The abundance of sly-mi398b decreased significantly 48 h after *B. cinerea* infection. Consistent with this, *CSD1* and *CSD2* transcripts were upregulated 48 hpi. These results suggest that the negative correlation between sly-miR398b, *CSD1* and *CSD2* affects tomato–*B. cinerea* interactions. Indeed, several studies have revealed that miR398 and its targets *CSD1/2* play crucial roles in disease resistance responses. For instance, in the Arabidopsis–*P. syringae* pv. tomato DC3000 interaction system, miR398b negatively regulates Arabidopsis defense, with the downregulation of miR398b and upregulation of *CSD1* upon infection [28,32]. In the common bean–*Sclerotinia sclerotiorum* interaction system, the downregulation of miR398b and an increase in *CSD1* transcripts have been observed upon infection. Moreover, the overexpression of miR398 promotes infection [43]. Recently, miR398b has been demonstrated to negatively regulate cotton immune responses to *Verticillium dahliae* via downregulating *GhCSD1*, *GhCSD2*, and *GhCCS* [44]. In contrast to the negative roles of miR398 in plant resistance in these studies, as well as in our work, many pieces of evidence have proposed an opposite role for miR398; for example, in rice against *M. oryzae* [35,37]. As for viruses, miR398 facilitates BMV accumulation in *N. benthamiana* [33], and it was speculated that miR398 enhances *N. benthamiana* resistance against the *beet necrotic yellow vein virus* in the same species [45]. Taken together, in response to various pathogens, miR398 has diverse regulatory mechanisms in plant immune responses. In plant–pathogen interactions, plants have evolved efficient mechanisms to combat pathogen attack. During pathogen infection, oxidative burst, hypersensitive response (HR), and ROS-induced cell death are considered to be major contributors to disease resistance [46]. Hypersensitive cell death is thought to inhibit infection by biotrophic pathogens; however, it facilitates the growth of necrotrophic pathogens, such as *B. cinerea* [46]. And indeed, *B. cinerea* infection results in ROS accumulation and triggers an HR in the affected tissue for its own benefit [47,48,49,50]. In the present study, hypersensitive cell death and increased H_2_O_2_ content were found in tomato leaves inoculated with *B. cinerea* 48 h prior. Compared to *PBI121#OE*, *sly-MIR398b#OE* leaves exhibited a higher H_2_O_2_ accumulation with larger lesion sizes, suggesting that higher H_2_O_2_ levels in *sly-MIR398b#OE* plants increase the susceptibility to *B. cinerea* compared to *PBI121#OE* control plants.

The phytohormone JA has long been known to positively regulate plant defenses against *B. cinerea* [6]. Mutants in the JA signaling pathway that display compromised resistance against *B. cinerea* are, for example, the JA receptor mutants *coi1-1* in Arabidopsis and *jai1* in tomato, which increased susceptibility to *B. cinerea* [5,6]. *TomLoxD* encodes a 13-lipoxygenase, which catalyzes a key step in JA biosynthesis [51,52]. The *TomLoxD* mutation in tomato increases susceptibility to *B. cinerea* infection, whereas *TomLoxD* overexpression has the opposite effect [7]. Additionally, *TomLoxD* was characterized as an early JA-responsive gene [5]. In tomato, LapA regulates defenses and wound signaling acting downstream of JA biosynthesis and perception [10]. This exopeptidase is induced by wounding and JA treatment and is known to mediate protein turnover during defense gene activation [53]. *PR-STH2* is a pathogen-responsive marker gene [5,54]. A previous study indicated that JA signaling is required for *B. cinerea* to induce the activation of *PR-STH2* in tomato [5]. Consistent with this, the transcript levels of *TomLoxD*, *LapA*, and *PR-STH2* were significantly upregulated in response to MeJA treatment and *B. cinerea* infection. Since the MeJA- and *B. cinerea*-induced expressions of these genes was significantly decreased in *sly-MIR398b#OE* plants compared with controls, we propose that the JA signaling pathway is involved in the sly-miR398b-mediated resistance against *B. cinerea* in tomato, and the decreased JA-responsive defense genes in *sly-MIR398b#OE* plants might contribute to their increased susceptibility to *B. cinerea*. JA modulates both plant growth and defense, and mediates the trade-offs between them [55,56]. Given the metabolic cost of sustained defense responses to plant growth, turning off JA signaling is of equal importance as turning it on. sly-miR398b might act as a built-in negative feedback regulation mechanism to avoid excessive JA-induced defense responses at the expense of plant growth. Of course, JA quantification in our experimental system might help to understand whether this subset of miR398 effects is due to altered JA synthesis and/or sensitivity. In addition to *sly-MIR398b* (located on chromosome 05), tomato contains two other *MIR398* members, *sly-MIR398a* (located on chromosome 11) and *sly-MIR398c* (located on chromosome 12). They encode three different mature sly-miR398, sly-miR398b (5′-uuguguucucaggucaccccu-3′), sly-miR398a (5′-uauguucucaggucgccccug-3′), and sly-miR398c (5′-uguguucucagguuaccccu-3′) [57,58]. The roles of sly-miR398a and sly-miR398c in tomato responses to *B. cinerea* infection also need to be tested in the future.

**Figure 7:**
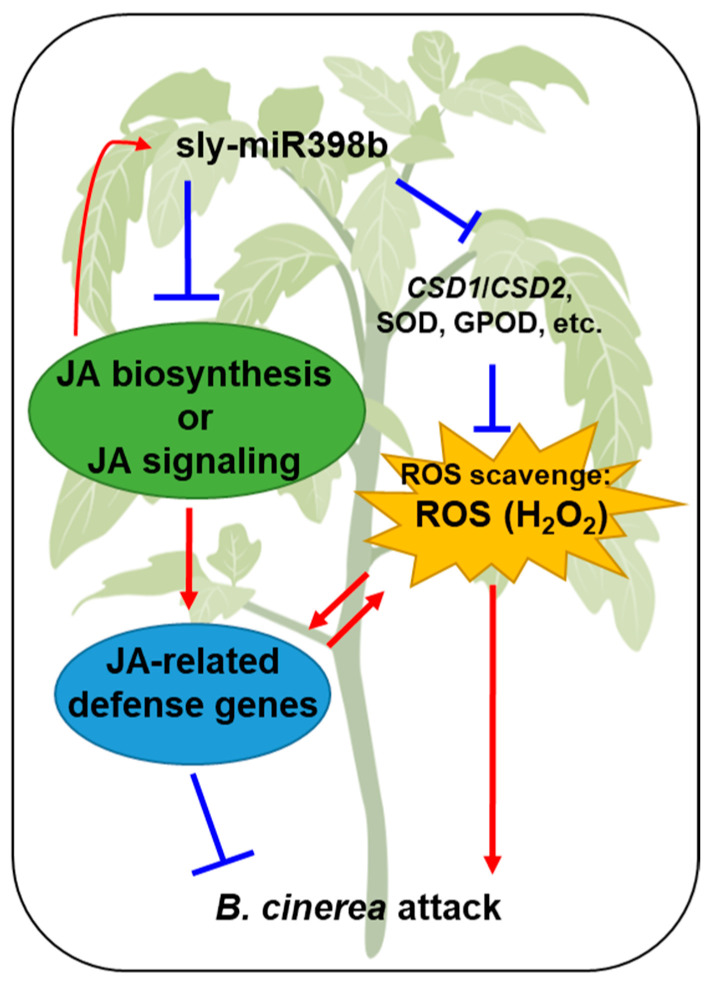
A schematic model of the role of sly-miR398b in promoting tomato susceptibility to *B. cinerea*. On the one hand, sly-miR398b overexpression downregulates *CSD1* and *CSD2*, as well as the activities of SOD and GPOD, which resulted in accumulated H_2_O_2_; on the other hand, it decreased JA-related defense genes, possibly by compromising JA biosynthesis or JA signaling.

Overall, the present study demonstrated that the overexpression of sly-miR398b negatively regulates the resistance to *B. cinerea* in tomato. These results reveal a new role of sly-miR398b in regulating tomato responses to *B. cinerea* by modulating ROS homeostasis and JA-responsive defense genes (Figure 7), and sly-miR398b might be a potential gene that can be applied for tomato resistance against *B. cinerea*. Further work in uncovering upstream regulators will help to elucidate the molecular mechanisms of sly-miR398b in suppressing tomato defense against *B. cinerea*, and the knockout mutant of sly-miR398b will also be required to fully unravel the role of sly-miR398b.

## 4. Materials and Methods

### 4.1. Plant Materials and Growth Conditions

Tomato (*Solanum lycopersicum* cv ‘Micro-Tom’) was used as the WT tomato plants. *pBI121#OE* (*pBI121* overexpressed control plants) and *sly-MIR398b#OE* (sly-miR398b overexpressing lines) plants were generated in ‘Micro-Tom’ background. Specifically, *sly-MIR398b* precursor (410 bp) was cloned from tomato and then inserted into vector *pBI121* (14,758 BP, a binary *Agrobacterium* vector with a *GUS* reporter gene for plant transformation) downstream of *CaMV 35S* promoter. The exact sequence length of *sly-MIR398b* precursor and the primer pair used for amplifying *sly-MIR398b* precursor sequence were included in the previous paper [38]. The resultant vector was then introduced into *Agrobacterium tumefaciens* strain GV3101 for tomato transformation. Homozygous transgenic plants were generated and identified as described previously [38]. Two sly-miR398b overexpressing lines (*MIR398b#OE1* and *MIR398b#OE8*) were obtained [38]. The amounts of sly-miR398b increased sharply to a similar extent in the two miR398b overexpressing lines (*MIR398b#OE1* and *MIR398b#OE8*) compared with those in control line, and they showed similar phenotypes with regard to the effects of salt stress on plant growth, oxidative damage, antioxidant response, and photosynthesis performance [38]. Therefore, in the present project, we selected one (*MIR398b#OE1*) of them (*MIR398b#OE1* and *MIR398b#OE8*) for further study. Tomato seedlings were grown in a growth chamber with temperature of 25 °C/20 °C day/night and photoperiod of 16/8 h light/dark (with a white light intensity of 200 µmol m^−2^ s^−1^).

### 4.2. B. cinerea Inoculation Assays

*B. cinerea* isolate B05.10 was cultured on potato dextrose agar for 14 d at 20 °C under a 12 h photoperiod prior to spore collection. Spore suspensions were prepared by harvesting the spores in 1% Sabouraud Maltose Broth, filtering them through four layers of medical gauze to remove hyphae, and adjusting the concentration to 1 × 10^6^ spores/mL. *B. cinerea* inoculation of tomato plants was performed as previously described [59], with minor modifications. To quantitatively measure lesion sizes, detached leaves from four-week-old tomato plants were placed in Petri dishes containing 0.8% agar medium (agar dissolved in sterile water), with the petiole embedded in the medium. Each leaflet was spotted with a single 5 µL droplet of *B. cinerea* spore suspension at a concentration of 1 × 10^6^ spores/mL. The trays were covered with lids and then kept in the growth chamber. Photographs were taken after 48 h, and the lesion sizes were recorded and calculated with Image J software [60]. For RT-qPCR experiments and antioxidant enzyme activity analysis, inoculations were performed in planta. Leaves of four-week-old plants were spotted with a 5 µL *B. cinerea* spore suspension (10^6^ spores/mL). To obtain saturating humidity conditions, the plants were covered with a clear plastic moisturizing lid and then incubated in the growth chamber. Spotted leaves were harvested 48 h later. In addition, leaves spotted with 1% Sabouraud Maltose Broth were considered uninfected leaves (mock). The leaf samples were frozen in liquid nitrogen and stored at −80 °C. To facilitate *B. cinerea* infection and tissue colonization, the light intensity in the growth chamber was set to 50 µmol m^−2^ s^−1^ for both in vitro and in planta *B. cinerea* inoculation assays.

### 4.3. RNA Extraction and Quantitative RT-PCR (qRT-PCR) Analysis

Total RNA was isolated from 0.1 g tomato leaves using TRIzol™ reagent (Invitrogen, Thermo Fisher Scientific, Waltham, MA, USA). For qRT-PCR, first strand cDNA was reverse transcribed from one microgram of DNA-free RNA with a PrimerScript II 1st Strand cDNA Synthesis Kit (TaKaRa). Oligo-dT primer and a miR398-specific primer were used to prepare the first cDNA strand of *mRNA* and miR398, respectively. The qRT-PCR was carried out with the TB Green Premix Ex Taq (TaKaRa) using UVP ChemStudio (Analytics Jena) following the protocol of 95 °C for 30 s, and 40 cycles at 95 °C for 5 s and 60 °C for 30 s. Levels of miR398b were normalized to the *U6* gene using the ΔCt method. Transcript abundance of all other loci was normalized to either one (*SlACTIN*: Solyc11g005330), or three reference genes (*SlACTIN*, *SlEF1α*: Solyc06g009970, *SlSAND*: Solyc03g115810) with the ΔCt method. Normalization factors were calculated as the geometric mean of their transcript levels when three reference genes were used [61]. Primers used referred to the previous paper [5,7,61] or were designed in this work with Primer-BLAST tool (http://www.ncbi.nlm.nih.gov/tools/primer-blast (accessed on 15 May 2022)), and they are listed in Supplementary Table S1. Each reaction was performed with three biological replicates, and each biological replicate contained three technical replicates. Each biological replicate consisted of the pooled leaves of four plants from one tray (different genotypes were grown together in a tray). Biological replicates were grown in different trays with different locations in the growth chambers and treated separately.

### 4.4. In Situ Staining and Measurement of O_2_^•−^ and H_2_O_2_

For detection of O_2_^•−^ and H_2_O_2_, inoculations were performed in planta as described in 4.2. Uninfected and *B. cinerea*-infected tomato leaves were collected 48 h after inoculation and stained with nitroblue tetrazolium (NBT) and 3,3-diaminobenzidine (DAB), respectively. Leaves were immersed in NBT solution (1 mg/mL, pH 7.4) or DAB solution (1 mg/mL, pH 3.8) followed by vacuum infiltration until they were completely infiltrated, and were then incubated for 3 h in the dark at room temperature. The NBT- and DAB-treated leaves were placed in 95% ethanol and kept in 65 °C water bath for 30 min to elute the chlorophyll. Subsequently, the accumulation of O_2_^•−^ and H_2_O_2_ in the leaves was captured with a digital camera.

### 4.5. Determination of Antioxidant Enzyme Activity

Uninfected and *B. cinerea*-infected tomato leaves were collected 48 h after inoculation to measure antioxidant enzyme activity. The activities of SOD, CAT, GPOD, and APX were determined using the methods described in a previous study [35], with some modifications. Briefly, 0.3 g of frozen leaves was ground with liquid nitrogen into powder. The frozen power was suspended in 2 mL of 50 mM phosphate-buffered saline (PBS, pH 7.8) for 5 min. The mixtures were centrifuged at 12,000× *g* for 20 min at 4 °C, and then supernatants were collected. The activity of SOD was calculated from its ability to inhibit the photochemical reduction in NBT. One unit of SOD activity was defined as the photochemical reduction that caused a 50% inhibition of NBT. The enzyme activity of CAT was defined by measuring the decrease in absorbance at 240 nm due to the decomposition of H_2_O_2_. The enzyme activity of GPOD was defined by calculating the increase in absorbance at 470 nm due to the polymerization of guaiacol to tetraguaiacol. The enzyme activity of APX was defined by calculating the decrease in absorbance at 290 nm due to ascorbate oxidation.

### 4.6. MeJA Treatment

Firstly, 18-day-old seedlings were kept in an airtight container, and then six cotton wicks that each contained 150 µL of 50 mM MeJA were placed evenly within the container. Tomato seedlings exposed to MeJA vapor were harvested at indicated time points and used for extracting total RNA [62].

## Data Availability

The data presented in this study are available in this manuscript.

## Supplementary Materials

The following supporting information can be downloaded at: https://www.mdpi.com/article/10.3390/plants12132572/s1, Table S1: Primers used in this study.
