# Supplementary material for: Overexpression of Sly-miR398b Compromises Disease Resistance against Botrytis cinerea through Regulating ROS Homeostasis and JA-Related Defense Genes in Tomato
Source: Plants (Basel). 2023 Jul 7;12(13):2572. doi: 10.3390/plants12132572 (PMC10346921; doi:10.3390/plants12132572)
Supplement: Supplementary file 1 [file plants-12-02572-s001.zip › plants-2400149-supplementary.pdf]

Supplementary Table S1. Primers used in this study.

Gene Name	Primer Sequence (5-3)
Pri-miRNA398b-Q-F	CTCATTTGTGTTGATAGAATGACC
Pri-miRNA398b-Q-R	GGGGATTCACCATTGCTCAT
sly-miRNA398b-RT-R	GTCGTATCCAGTGCAGGGTCCGAGGTATTCGCACTGG ATACGACAGGGGT
sly-miRNA398b-Q-F	GCGCGTTGTGTTCTCAGGTC
sly-miRNA398b-Q-R	AGTGCAGGGTCCGAGGTATT
U6-Q-F	CATCCGATAAAATTGGAACGA
U6-Q-R	TTTGTGCGTGTCATCCTTGCG
SlACTIN-Q-F	TTGCTGACCGTATGAGCAAG
SlACTIN-Q-R	GGACAATGGATGGACCAGAC
SlEF1α-Q-F	TACTGGTGGTTTTGAAGCTG
SlEF1α-Q-R	AACTTCCTTCACGATTTCATCATA
SlSAND-Q-F	TTGCTTGGAGGAACAGACG
SlSAND-Q-R	GCAAACAGAACCCCTGAATC
CSD1-Q-F	GGTGTTAGTGGCACCATCCT
CSD1-Q-R	AGCACCATGCTCCTTACCAG
CSD2-Q-F	ACATTGTTGCTGGTCCTAATGAGA
CSD2-Q-R	CCCAATAACGCCTCTTCCCA
CAT-Q-F	TACGGTTGGTGCAAGAGGTC
CAT-Q-R	CAGCACAGGTAAGGTGAGCA
POD-Q-F	TCGAACAAAGAAAAGCAGCTC
POD-Q-R	TGACCATAGCTAGAACCCGC
APX-Q-F	GCTGACTTGGTTCAGTTGGC
APX-Q-R	AGCCTTCCCTCTTCTGGACA
TomLoxD-Q-F	ACTCATCAGCACCGACATCG
TomLoxD-Q-R	ACTCTCCAGAAAGAACTCCTGC
LapA-Q-F	ATCTCAGGTTTCCTGGTGGAAGGA
LapA-Q-R	AGTTGCTATGGCAGAGGCAGAG
PR-STH2-Q-F	GAAGGGGATCCATTGGGACAA
PR-STH2-Q-R	TTCCCATAGCACTATCTTTTCCA
